# Channel Covariance Matrix Estimation via Dimension Reduction for Hybrid MIMO MmWave Communication Systems

**DOI:** 10.3390/s19153368

**Published:** 2019-07-31

**Authors:** Rui Hu, Jun Tong, Jiangtao Xi, Qinghua Guo, Yanguang Yu

**Affiliations:** School of Electrical, Computer and Telecommunications Engineering, University of Wollongong, Wollongong, NSW 2522, Australia

**Keywords:** millimeter wave communications, hybrid MIMO, channel covariance estimation

## Abstract

Hybrid massive MIMO structures with lower hardware complexity and power consumption have been considered as potential candidates for millimeter wave (mmWave) communications. Channel covariance information can be used for designing transmitter precoders, receiver combiners, channel estimators, etc. However, hybrid structures allow only a lower-dimensional signal to be observed, which adds difficulties for channel covariance matrix estimation. In this paper, we formulate the channel covariance estimation as a structured low-rank matrix sensing problem via Kronecker product expansion and use a low-complexity algorithm to solve this problem. Numerical results with uniform linear arrays (ULA) and uniform squared planar arrays (USPA) are provided to demonstrate the effectiveness of our proposed method.

## 1. Introduction

Millimeter wave (mmWave) communications are promising for future-generation wireless communications for their advantages such as large bandwidths, narrow beams, and secure transmissions [1,2]. Large-scale multiple-input multiple-output (MIMO) hybrid structures equipped with only a few RF chains have generated great interests for mmWave systems due to their low complexity and near-optimal performance [3,4]. Precoders and combiners must be carefully designed to exploit the potential of large-scale MIMO hybrid systems, e.g., to achieve high data transmission rates. They can be designed based on the instantaneous channel matrix [5,6], which may be estimated by using channel estimation techniques [3,7]. However, the instantaneous channel can vary fast [8], especially at mmWave frequencies [9,10], and the precoder/combiner has to be redesigned once the instantaneous channel changes [5,6].

Although the instantaneous mmWave channel can change very fast, the long-term channel statistics, e.g., the angular power spectrum, can be stationary for tens to hundreds of coherence blocks [9]. Recently, the channel covariance information has been utilized to design the analog precoders/combiners [9,11], which remain fixed when the covariance matrix is unchanged. The effective digital system has a reduced dimensionality, which greatly reduces the cost of acquiring the instantaneous channel state information (CSI) and simplifies the optimization of the digital precoders and combiners. The channel covariance matrix should be firstly estimated to realize the designs in [9,11]. With large antenna arrays, the channel covariance matrix has a large dimensionality, which demands a large number of observations to be used when traditional covariance matrix estimators are adopted. Meanwhile, the hybrid structure only allows a reduced number of observations to be acquired at the receiver, which makes the task of channel covariance estimation challenging. In order to address this challenge, [10,12] propose several compressive sensing (CS) based channel covariance estimators, which explore the relations between the angle of departure (AoD)/angle of arrival (AoA) and the channel covariance matrix. Their methods need a dictionary for searching the AoD/AoA, and the resulting performance improves when the resolution of the dictionary becomes higher. However, high-resolution dictionary yields high computational complexity. Moreover, these CS-based estimators require the number of paths in the channel to be known a priori. In [13], an analytical expression of the channel covariance matrix is derived and computed through the information obtained from one instantaneous channel realization, which can be estimated from low-dimensional observations. In [14], the covariance matrices of vector channels are estimated by solving a subspace estimation problem leveraging their low-rank property. Also, tensor decomposition has been used for dimension reduction for the mmWave channel estimation problem [15,16]. It has been recently used for channel covariance estimation in frequency-selective channels [17], where the channel is represented as a low-rank third-order tensor in terms of factor matrices. The channel covariance matrix is obtained from the estimated factor matrices. The methods of [13,14,17] focus on vector channels, which may not be directly applicable to matrix channels where both the transmitter and receiver employ multiple antennas.

In this paper, we investigate the mmWave channel covariance matrix estimation problem for hybrid mmWave communication systems that are equipped with uniform linear arrays (ULA) or uniform square planar arrays (USPA). Both the transmitter and the receiver have multiple antennas. The main contributions are as follows:

1. We show that the mmWave MIMO channel covariance matrix follows a Kronecker product expansion model [18]. Following [18,19,20], we show that this model can be used for reducing the effective dimension of the large-dimensional channel covariance matrices in mmWave MIMO systems. We further show that permutation can reduce the rank of the mmWave channel covariance matrix, which admits an expression of the summation of vector outer products. We thus formulate the channel covariance matrix estimation problem as a low-rank matrix sensing problem.
2. Although the aforementioned low-rank matrix sensing problem has a smaller size than the original problem, the complexity can still be high when the numbers of the transmitter/receiver antennas are large. In order to reduce the complexity, we further exploit the structures of the ULA or USPA to reduce the dimensionality of the problem and formulate the problem as a *structured* low-rank matrix sensing problem. We adapt the recently proposed generalized conditional gradient and alternating minimization (GCG-Alt) algorithm [21], which has low computational complexity, to find the solution. Numerical results with ULA and USPA suggest that our proposed estimator is effective in estimating the mmWave channel covariance matrix.

The rest of this paper is organized as follows. We introduce the spatial channel model and the hybrid system in Section 2. In Section 3, we formulate the channel covariance estimation problem as a structured low-rank matrix sensing problem and present the solution. We show the simulation results in Section 4 and conclude the paper in Section 5.

Notations: Bold uppercase A denotes a matrix and bold lowercase a denotes a column vector. $A^{*}$, $A^{T}$, and $A^{H}$ denote the conjugate, transpose, and conjugate transpose of matrix A, respectively. a(i) denotes the *i*-th element of vector a. ${\left[A\right]}_{a:b,:}$ denotes the submatrix of A made of its *a*-th to *b*-th rows. ${\left[A\right]}_{a:b,c:d}$ denotes the submatrix of A defined by its *a*-th to *b*-th rows and *c*-th to *d*-th columns. ${∥A∥}_{F}$ and ${∥A∥}_{*}$ are the Frobenius norm and the nuclear norm of A. For $A\in C^{M\times N}$, $\mathrm{vec}\left(A\right)\in C^{MN\times 1}$ is a column vector obtained through the vectorization of A and ${\mathrm{vec}}^{-1}\left(A\right)\in C^{M\times N}$ is a matrix obtained by the inverse of vectorization. For matrices A and B, A⊗B denotes the Kronecker product of A and B. $\mathrm{CN}(a,b^{2})$ represents complex Gaussian distribution with mean *a* and variance $b^{2}$. U(a,b) represents uniform distribution with support [a,b].

## 2. Spatial Channel Model

Consider point-to-point mmWave transmissions, where the transmitter has $N_{t}$ antennas and the receiver has $N_{r}$ antennas. We assume the following spatial channel [22]:

(1) $$H=\frac{1}{\sqrt{L}}\sum_{k=1}^{K}\sum_{l=1}^{L}g_{kl}a_{r}\left(\phi_{kl}^{r},\theta_{kl}^{r}\right)a_{t}^{H}\left(\phi_{kl}^{t},\theta_{kl}^{t}\right)\in C^{N_{r}\times N_{t}},$$

where *K* is the number of clusters, and *L* is the number of rays within each cluster. As reported in [22], the number of clusters is often small, e.g., K=1,2, but the number of rays inside each cluster can be large, e.g., L=30. $a_{r}\left(\phi_{kl}^{r},\theta_{kl}^{r}\right)$ and $a_{t}\left(\phi_{kl}^{t},\theta_{kl}^{t}\right)$ are the array response vectors at the receiver and transmitter, respectively, where $\phi_{kl}^{r},\theta_{kl}^{r},\phi_{kl}^{t}$, and $\theta_{kl}^{t}$ are the azimuth AoA, elevation AoA, azimuth AoD, and elevation AoD on the *l*-th ray of the *k*-th cluster, respectively. These angles can be characterized by cluster center angles and angular spreads: Each cluster covers a range of angles and the angular spread describes the span of each cluster. The angular spread in the mmWave propagation environment is considered to be small [13]. Measurements of the angular spread taken in the urban area of New York City are presented in [22] in terms of the root-mean-square (rms) of all the measurements. At the carrier frequency $f_{c}=28$ GHz, example angular spreads of $15.5^{\circ },6^{\circ },10.2^{\circ }$, and $0^{\circ }$ are reported for $\phi_{kl}^{r},\theta_{kl}^{r},\phi_{kl}^{t}$, and $\theta_{kl}^{t}$, respectively. The small-scale fading coefficient $g_{kl}$ is assumed complex Gaussian, i.e., $g_{kl}∼\mathrm{CN}\left(0,\gamma_{k}^{2}\right)$, where $\gamma_{k}^{2}$ is the fraction power of the *k*-th cluster [22] (Equation (7)).

As discussed in [9], though the small-scale fading gains $\left\{g_{kl}\right\}$ change fast, the AoDs/AoAs and $\gamma_{k}^{2}$ may remain stationary over tens to hundreds of coherence blocks. Assume that $\left\{g_{kl},\forall k,\forall l\right\}$ are mutually independent, then channel covariance matrix can be modeled as

(2) $$\begin{array}{cc} R & ≜E[\mathrm{vec}\left(H\right){\mathrm{vec}}^{H}\left(H\right)]\\ & =\frac{1}{L}\sum_{k=1}^{K}\gamma_{k}^{2}\sum_{l=1}^{L}{\tilde{T}}_{kl}^{t}⊗{\tilde{T}}_{kl}^{r}\in C^{N_{r}N_{t}\times N_{r}N_{t}}, \end{array}$$

where

(3) $${\tilde{T}}_{kl}^{t}≜a_{t}^{*}\left(\phi_{kl}^{t},\theta_{kl}^{t}\right)a_{t}^{T}\left(\phi_{kl}^{t},\theta_{kl}^{t}\right)\in C^{N_{t}\times N_{t}},$$

and

(4) $${\tilde{T}}_{kl}^{r}≜a_{r}\left(\phi_{kl}^{r},\theta_{kl}^{r}\right)a_{r}^{H}\left(\phi_{kl}^{r},\theta_{kl}^{r}\right)\in C^{N_{r}\times N_{r}}.$$

Note that Equation (2) is the same as the channel covariance expression in [9] when L=1. In the following, we first present our proposed covariance matrix estimation method for systems equipped with the ULA and then discuss its adaptation to systems that adopt the USPA.

For the ULA, the array responses $a_{t}\left(\phi_{kl}^{t},\theta_{kl}^{t}\right)$ and $a_{r}\left(\phi_{kl}^{r},\theta_{kl}^{r}\right)$ are independent of the elevation angles. They can thus be abbreviated as $a_{t}\left(\phi_{kl}^{t}\right)$ and $a_{r}\left(\phi_{kl}^{r}\right)$. For an $N_{a}$-element ULA with distance *d* between adjacent antennas, the array response is

$$a\left(\phi_{kl}\right)=\frac{1}{\sqrt{N_{a}}}{\left[1,e^{j\frac{2\pi }{\lambda_{c}}d\sin\left(\phi_{kl}\right)},\cdots ,e^{j\left(N_{a}-1\right)\frac{2\pi }{\lambda_{c}}d\sin\left(\phi_{kl}\right)}\right]}^{T},$$

where $\lambda_{c}$ is the carrier wavelength and $N_{a}=N_{t}$ or $N_{r}$ is the number of antennas at the transmitter or receiver. Accordingly, ${\tilde{T}}_{kl}^{t}$ of Equation (3) and ${\tilde{T}}_{kl}^{r}$ of Equation (4) become

(5) $${\tilde{T}}_{kl}^{t}=a_{t}^{*}\left(\phi_{kl}^{t}\right)a_{t}^{T}\left(\phi_{kl}^{t}\right)$$

and

(6) $${\tilde{T}}_{kl}^{r}=a_{r}\left(\phi_{kl}^{t}\right)a_{r}^{H}\left(\phi_{kl}^{t}\right),$$

respectively, which are Toeplitz–Hermitian. Since the Kronecker product of two Toeplitz–Hermitian matrices is block-Toeplitz–Hermitian [23], the channel covariance matrix R defined in Equation (2) is block-Toeplitz–Hermitian.

**Figure 1:**
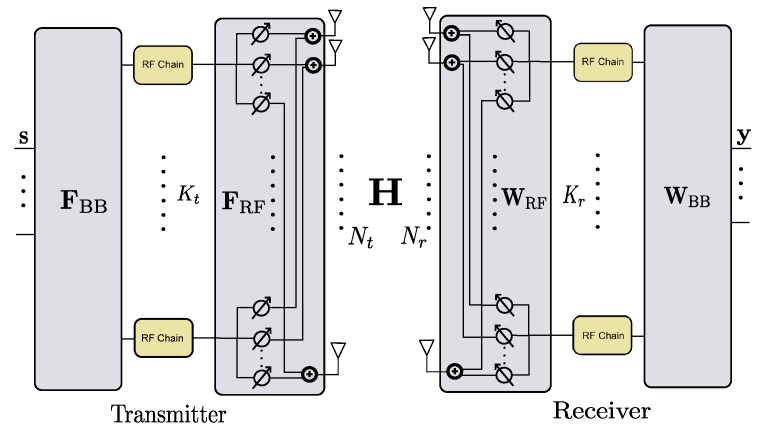
The phase shifter-based hybrid transceiver.

We next discuss the hybrid system. We assume phase shifter-based hybrid transceivers [21] shown in Figure 1, where the antennas and analog phase shifters at the transmitter or receiver are fully connected. Assume that there are $K_{t}\ll N_{t}$ radio frequency (RF) chains at the transmitter and $K_{r}\ll N_{r}$ RF chains at the receiver. For single-stream transmissions with one symbol *s* transmitted, the received signal is written as

(7) $$y=W^{H}Hfs+W^{H}n,$$

where W and f are the receiving processing matrix and transmitting processing vector, respectively, and n is the noise vector. Up to $K_{r}$ digital symbols can be observed at the receiver after each transmission. In hybrid transceivers, we have $W=W_{\mathrm{RF}}W_{\mathrm{BB}}$ and $f=F_{\mathrm{RF}}f_{\mathrm{BB}}$, where $W_{\mathrm{RF}}$ and $F_{\mathrm{RF}}$ are the analog combiner and precoder, respectively, and $W_{\mathrm{BB}}$ and $f_{\mathrm{BB}}$ are the digital combiner and precoder, respectively. In addition, due to the constraints of the phase shifters in the RF combiner and precoder, the entries in $W_{\mathrm{RF}}$ and $F_{\mathrm{RF}}$ have constant modulus.

Note that using single-stream transmissions during the channel training avoids the interferences caused by transmitting multiple symbols simultaneously, and this has been widely considered [3,4,7].

When $N_{t}$ and $N_{r}$ are large, the dimension of the channel covariance matrix R is large. In this case, estimating R can be difficult when only a small number of observations available, which is typical in the hybrid system.

From Equation (2), R follows the Kronecker product expansion model [18]. In the following, we explore this property and the block-Toeplitz–Hermitian structure of R to reduce the dimensionality of the problem of estimating R, and formulate the channel covariance matrix estimation problem as a structured low-rank matrix sensing problem.

## 3. Structured Low-Rank Covariance Matrix Sensing

### 3.1. Rank Reduction By Permutation

Define

(8) $$T_{kl}^{t}=\frac{\gamma_{k}}{\sqrt{L}}{\tilde{T}}_{kl}^{t}\in C^{N_{t}\times N_{t}}$$

and

(9) $$T_{kl}^{r}=\frac{\gamma_{k}}{\sqrt{L}}{\tilde{T}}_{kl}^{r}\in C^{N_{r}\times N_{r}}$$

respectively, where 1≤l≤L and 1≤k≤K. Then R of Equation (2) can be written compactly as

(10) $$R=\sum_{k=1}^{K}\sum_{l=1}^{L}T_{kl}^{t}⊗T_{kl}^{r}\in C^{N_{t}N_{r}\times N_{t}N_{r}},$$

where the summation involves KL terms. Note that $T_{kl}^{t}$ and $T_{kl}^{r}$ are Toeplitz–Hermitian. Denote the following $N_{r}\times N_{r}$ submatrix of R as

(11) $$R_{mn}≜{\left[R\right]}_{\left(\left(m-1\right)N_{r}+1\right):mN_{r},\left(\left(n-1\right)N_{r}+1\right):nN_{r}},$$

where $1\leq m\leq N_{t}$ and $1\leq n\leq N_{t}$. Define a permutation operator P(·) that permutes the $N_{t}N_{r}\times N_{t}N_{r}$ matrix R into a $N_{t}^{2}\times N_{r}^{2}$ matrix

$$R_{p}=P\left(R\right)$$

by stacking each submatrix $R_{mn}$ into a row vector as

$${\left[P\left(R\right)\right]}_{m+\left(n-1\right)N_{t},:}={\mathrm{vec}}^{T}\left(R_{mn}\right)\in C^{1\times N_{r}^{2}}.$$

We write

$$t_{kl}^{t}=\mathrm{vec}\left(T_{kl}^{t}\right)\in C^{N_{t}^{2}\times 1},$$

and

$$t_{kl}^{r}=\mathrm{vec}\left(T_{kl}^{r}\right)\in C^{N_{r}^{2}\times 1}.$$

Then based on the Kronecker product expansion property [23,24], $R_{p}$ can be written as a sum of vector outer products

(12) $$R_{p}=\sum_{k=1}^{K}\sum_{l=1}^{L}t_{kl}^{t}{\left(t_{kl}^{r}\right)}^{T}\in C^{N_{t}^{2}\times N_{r}^{2}}.$$

Note that if we have $R_{p}$, we can obtain R as $P^{-1}\left(R_{p}\right)$.

From Equation (12), we can see that the column space of $R_{p}$ is spanned by $\left\{t_{kl}^{t}\right\}$ and the row space of $R_{p}$ is spanned by $\left\{t_{kl}^{r}\right\}$. Recall that $t_{kl}^{t}=\mathrm{vec}\left(T_{kl}^{t}\right)$ and by using the relation between $T_{kl}^{t}$ and the transmitter array response $a_{t}\left(\phi_{kl}^{t}\right)$ shown in Equations (5) and (8), $t_{kl}^{t}$ can be written as

(13) $$\begin{array}{cc} t_{kl}^{t} & =\frac{\gamma_{k}}{\sqrt{N_{t}L}}{\left[a_{t}^{T}\left(\phi_{kl}^{t}\right),e^{-j\frac{2\pi }{\lambda_{c}}d\sin\left(\phi_{kl}^{t}\right)}a_{t}^{T}\left(\phi_{kl}^{t}\right),\ldots ,e^{-j\left(N_{t}-1\right)\frac{2\pi }{\lambda_{c}}d\sin\left(\phi_{kl}^{t}\right)}a_{t}^{T}\left(\phi_{kl}^{t}\right)\right]}^{T}\\ & =\frac{\gamma_{k}}{\sqrt{L}}a_{t}^{*}\left(\phi_{kl}^{t}\right)⊗a_{t}\left(\phi_{kl}^{t}\right), \end{array}$$

where $\phi_{kl}^{t}$ is the azimuth AoD. We can see that $t_{kl}^{t}$ consists of the array response vector $a_{t}\left(\phi_{kl}^{t}\right)$ and the column space of $R_{p}$ is determined by the set

$$C_{t}=\left\{a_{t}^{*}\left(\phi_{kl}^{t}\right)⊗a_{t}\left(\phi_{kl}^{t}\right),1\leq k\leq K,1\leq l\leq L\right\}.$$

As introduced earlier, small angular spreads are observed in the mmWave propagation environment, which indicates that the AoDs inside a cluster are closely spaced and their corresponding array response vectors are highly correlated. Therefore, for the *k*-th cluster, though the number of rays *L* inside can be large, the space spanned by $\left\{a_{t}^{*}\left(\phi_{kl}^{t}\right)⊗a_{t}\left(\phi_{kl}^{t}\right),1\leq l\leq L\right\}$ may be well approximated by a low-rank space. In addition, since the number of clusters *K* is generally small (e.g., K=1 or 2), both $C_{t}$ and R can be low-rank. This is similar to the low-rankness of the mmWave channel H, which has been validated by the experimental and simulation results in [22]. The low-rank property of $R_{p}$ can be shown numerically. Denote by $r_{\mathrm{ch}}$ the rank of $R_{p}$ or R, and let $\sigma_{1}>\sigma_{2}>\ldots >\sigma_{r_{\mathrm{ch}}}$ be the singular values of $R_{p}$ or R. We may use

(14) $$p_{e}\overset{\Delta }{=}\frac{\sum_{j=1}^{r_{\mathrm{sub}}}\sigma_{j}^{2}}{\sum_{i=1}^{r_{\mathrm{ch}}}\sigma_{i}^{2}}$$

to measure the energy captured by a rank-$r_{\mathrm{sub}}$ approximation of $R_{p}$ or R, where $r_{\mathrm{sub}}$ is the rank of the subspace of $R_{p}$ or R.

**Figure 2:**
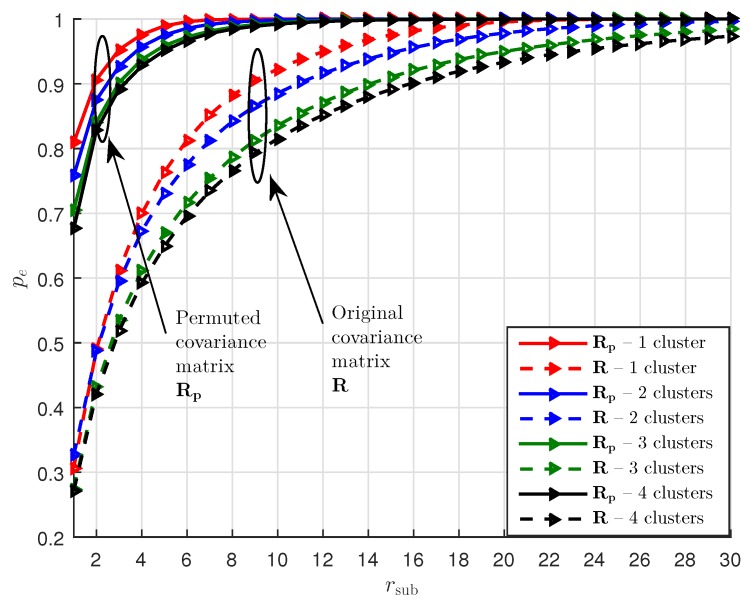
Energy captured by a rank-$r_{\mathrm{sub}}$ approximation of $R_{p}$ and R.

Figure 2 shows an example of a ULA system with K∈{1,2,3,4},L=30, $N_{t}=64$, and $N_{r}=16$. The covariance matrix R and its permuted version $R_{p}$ have sizes of 1024×1024 and 4096×256, respectively, which shows that $R_{p}$ is a taller matrix. The horizontal AoDs

(15) $$\phi_{kl}^{t}∼U\left(\phi_{k}^{t}-\upsilon_{h}^{t},\phi_{k}^{t}+\upsilon_{h}^{t}\right),l=1,2,\cdots ,L,$$

where the center angles $\phi_{k}^{t}$ are distributed uniformly in [0,2π] and separated by at least one angular spread $\upsilon_{h}^{t}=10.2^{\circ }$. Similarly, the horizontal AoAs

(16) $$\phi_{kl}^{r}∼U\left(\phi_{k}^{r}-\upsilon_{h}^{r},\phi_{k}^{r}+\upsilon_{h}^{r}\right),l=1,2,\cdots ,L,$$

where $\upsilon_{h}^{r}=15.5^{\circ }$.

The cluster powers are generated following [22] (Table I). It can be seen from Figure 2 that for capturing a majority of the total energy, e.g., with $p_{e}=0.95,0.99$, the required $r_{\mathrm{sub}}$ for $R_{p}$ is generally much smaller than $\min(N_{t}^{2},N_{r}^{2},KL)$ and is also much smaller than that for R. In the following, we use $r_{p}$ as the $r_{\mathrm{sub}}$ of $R_{p}$ and $r_{R}$ as the $r_{\mathrm{sub}}$ of R for a certain $p_{e}$. As such, $R_{p}$ may be well approximated as a rank-$r_{p}$ matrix. One may use low-rank matrix recovery methods, e.g., matrix completion methods, to estimate the best rank-$r_{p}$ approximation of $R_{p}$ from a small amount of observations. However, when $N_{t}$ and $N_{r}$ are large, which is the case in mmWave communications, the number of parameters required by the rank-$r_{p}$ approximation of $R_{p}$, i.e., $\left(N_{t}^{2}+N_{t}^{2}\right)\times r_{p}$, is still large. Therefore, estimating the subspaces of $R_{p}$ can be computationally expensive.

### 3.2. Dimension Reduction by Exploiting the Toeplitz–Hermitian Structure

Recall that R is block-Toeplitz–Hermitian and $R_{p}=P\left(R\right)$ is a permutation of R. From Equations (12) and (13), we can see that $R_{p}$ is also specially structured: $R_{p}$ is the summation of the outer products of $t_{kl}^{t}$ and $t_{kl}^{r}$, where $t_{kl}^{t}$ and $t_{kl}^{r}$ are the vectorizations of Toeplitz–Hermitian matrices $T_{kl}^{t}$ and $T_{kl}^{r}$, respectively. Since the Toeplitz–Hermitian matrix $T_{kl}^{t}\in C^{N_{t}^{2}\times N_{t}^{2}}$ is determined by its first column and first row (its first row is the conjugate transpose of its first column), we can represent $t_{kl}^{t}$ in terms of the entries in the first column and first row of $T_{kl}^{t}$. We can represent $t_{kl}^{r}$ in the same way. Therefore, the total numbers of unknowns in $t_{kl}^{t}$ and $t_{kl}^{r}$ are $2N_{t}-1$ and $2N_{r}-1$, respectively. Then we can reduce the problem size of $\left(N_{t}^{2}+N_{r}^{2}\right)\times r_{p}$ to $2\left(N_{t}+N_{r}-1\right)\times r_{p}$. In the following, we show how the problem size can be reduced.

First, let us use an example with $N_{t}=3$ to illustrate the structure of $t_{kl}^{t}$. The array response

$$a_{t}\left(\phi_{kl}^{t}\right)=\frac{1}{\sqrt{3}}{\left[1,e^{j\frac{2\pi }{\lambda_{c}}d\sin\left(\phi_{kl}^{k}\right)},e^{j2\frac{2\pi }{\lambda_{c}}d\sin\left(\phi_{kl}^{k}\right)}\right]}^{T}.$$

Then according to Equation (13), we have

$$t_{kl}^{t}=\frac{\sqrt{L}}{\gamma_{k}}a_{t}^{*}\left(\phi_{kl}^{t}\right)⊗a_{t}\left(\phi_{kl}^{t}\right)=\frac{\sqrt{L}}{3\gamma_{k}}\left[\begin{array}{c} 1\\ e^{j\frac{2\pi }{\lambda_{c}}d\sin\left(\phi_{kl}^{k}\right)}\\ e^{j2\frac{2\pi }{\lambda_{c}}d\sin\left(\phi_{kl}^{k}\right)}\\ e^{-j\frac{2\pi }{\lambda_{c}}d\sin\left(\phi_{kl}^{k}\right)}\\ 1\\ e^{j\frac{2\pi }{\lambda_{c}}d\sin\left(\phi_{kl}^{k}\right)}\\ e^{-j2\frac{2\pi }{\lambda_{c}}d\sin\left(\phi_{kl}^{k}\right)}\\ e^{-j\frac{2\pi }{\lambda_{c}}d\sin\left(\phi_{kl}^{k}\right)}\\ 1 \end{array}\right].$$

We can see that all the 9 elements in $t_{kl}^{t}$ can be represented by the elements in $a_{t}\left(\phi_{kl}^{t}\right)$ and $a_{t}^{*}\left(\phi_{kl}^{t}\right)$. Now construct a vector

$$a_{kl}=\frac{\sqrt{L}}{3\gamma_{k}}{\left[a_{t}^{T}\left(\phi_{kl}^{t}\right),e^{-j\frac{2\pi }{\lambda_{c}}d\sin\left(\phi_{kl}^{k}\right)},e^{-j2\frac{2\pi }{\lambda_{c}}d\sin\left(\phi_{kl}^{k}\right)}\right]}^{T}\in C^{5\times 1},$$

then $t_{kl}^{t}$ can be rewritten as

$$t_{kl}^{t}=\left[\begin{array}{c} \left[I_{3},0_{3\times 2}\right]\\ 0_{1\times 3},1,0]\\ I_{2},0_{2\times 3}]\\ 0_{1\times 4},1]\\ 0_{1\times 3},1,0]\\ 0_{1\times 4}] \end{array}\right]a_{kl}.$$

In fact, $a_{kl}\left(4\right)={\left(a_{kl}\left(2\right)\right)}^{*}$ and $a_{kl}\left(5\right)={\left(a_{kl}\left(3\right)\right)}^{*}$. Therefore, $t_{kl}^{t}$ can be expressed as a product of a weight matrix and a vector $a_{kl}$. Furthermore, the weight matrix depends only on the structure of the antenna array and is independent of the path angles.

Similarly, for the general cases, we can express $t_{kl}^{t}$ with a weight matrix $\Gamma_{u}\in C^{N_{t}^{2}\times \left(2N_{t}-1\right)}$ and a vector $a_{kl}\in C^{(2N_{t}-1)\times 1}$, and express $t_{kl}^{r}$ with a weight matrix $\Gamma_{v}\in C^{N_{r}^{2}\times \left(2N_{r}-1\right)}$ and a vector $b_{kl}\in C^{(2N_{r}-1)\times 1}$. We require

$$a_{kl}\left(x+N_{t}-1\right)={\left(a_{kl}\left(x\right)\right)}^{*},2\leq x\leq N_{t},$$

and

$$b_{kl}\left(y+N_{r}-1\right)={\left(b_{kl}\left(y\right)\right)}^{*},2\leq y\leq N_{r}.$$

We then have

(17) $$t_{kl}^{t}=\Gamma_{u}a_{kl},\;\mathrm{and}\;t_{kl}^{r}=\Gamma_{v}b_{kl},$$

where $\Gamma_{u}=\left[\Gamma_{u1},\Gamma_{u2}\right]$ with $\Gamma_{u1}\in C^{N_{t}^{2}\times N_{t}}$, $\Gamma_{u2}\in C^{N_{t}^{2}\times \left(N_{t}-1\right)}$, and

$$\Gamma_{u1}=\left[\begin{array}{c} I_{N_{t}}\\ 0_{1\times N_{t}}\\ I_{N_{t}-1},0_{(N_{t}-1)\times 1}]\\ 0_{2\times N_{t}}\\ I_{N_{t}-2},0_{(N_{t}-2)\times 2}]\\ 0_{3\times N_{t}}\\ \vdots \\ 0_{1\times (N_{t}-1)}] \end{array}\right],\;\Gamma_{u2}=\left[\begin{array}{c} 0_{N_{t}\times \left(N_{t}-1\right)}\\ e_{1}^{T}\\ 0_{N_{t}-1\times \left(N_{t}-1\right)}\\ e_{2}^{T}\\ e_{1}^{T}\\ 0_{\left(N_{t}-2\right)\times \left(N_{t}-1\right)}\\ e_{3}^{T}\\ e_{2}^{T}\\ e_{1}^{T}\\ \vdots \\ 0_{1\times (N_{t}-1)} \end{array}\right]$$

with $e_{i}\in C^{(N_{t}-1)\times 1}$ being a vector whose *i*-th entry is 1 and other entries are zero. $\Gamma_{v}$ is constructed similarly as $\Gamma_{u}$, and $\Gamma_{u}$ and $\Gamma_{v}$ are both full-rank. This is because $\Gamma_{u}$ and $\Gamma_{v}$ consist of 1’s and 0’s, and there is only one 1 in each row of $\Gamma_{u}$ and $\Gamma_{v}$. Therefore, Equation (12) can be rewritten as

(18) $$\begin{array}{cc} R_{p} & =\sum_{k=1}^{K}\sum_{l=1}^{L}\Gamma_{u}a_{kl}b_{kl}^{T}\Gamma_{v}^{T}\\ & =\Gamma_{u}\left(\sum_{k=1}^{K}\sum_{l=1}^{L}a_{kl}b_{kl}^{T}\right)\Gamma_{v}^{T}\\ & =\Gamma_{u}C\Gamma_{v}^{T}, \end{array}$$

where $C=\sum_{k=1}^{K}\sum_{l=1}^{L}a_{kl}b_{kl}^{T}$.

As shown above, $R_{p}$ is approximately low-rank. Since the fixed weight matrices $\Gamma_{u}$ and $\Gamma_{v}$ are full-rank, C is approximately low-rank. Hence estimating a low-rank approximation of $R_{p}$ is equivalent to estimating a low-rank approximation of C. Note that the size of $C\in C^{\left(2N_{t}-1\right)\times \left(2N_{r}-1\right)}$ is much smaller than the size of $R_{p}\in C^{N_{t}^{2}\times N_{r}^{2}}$, and this can greatly reduce the complexity of the problem.

### 3.3. Training

We assume that the channel matrix H remains static during a snapshot and suppose we have *T* snapshots. For different snapshots, we assume that the AoAs/AoDs and the fraction power $\gamma_{k}^{2}$ remain unchanged, but the small-scale fading gain $g_{kl}∼\mathrm{CN}\left(0,\gamma_{k}^{2}\right)$ can change [9]. Suppose the transmitter sends out *S* training beams during each snapshot. For the *s*-th training beam of the *t*-th snapshot, we employ the transmitting vector $f_{t,s}\in C^{N_{t}}$ and the receiving matrix $W_{t,s}\in C^{N_{r}\times K_{r}}$. Therefore, in each snapshot, after the transmitter sends out *S* training beams, the receiver receives $SK_{r}$ symbols, and the sampling ratio is $SK_{r}/N_{r}N_{t}$. We design $f_{t,s}$ and $W_{t,s}$ and their corresponding $F_{\mathrm{RF}},f_{\mathrm{BB}},W_{\mathrm{RF}}$, and $W_{\mathrm{BB}}$ realizations for the hybrid structure according to the training scheme in [21] (Section III.D). For the *s*-th training beam of the *t*-th snapshot, the received signal is

(19) $$\begin{array}{cc} y_{t,s} & =W_{t,s}^{H}H_{t}f_{t,s}s+W_{t,s}^{H}n_{t,s}\\ & =\left(f_{t,s}^{T}⊗W_{t,s}^{H}\right)\mathrm{vec}\left(H_{t}\right)s+W_{t,s}^{H}n_{t,s}, \end{array}$$

where $n_{t,s}$ is the noise vector and $H_{t}$ is the channel matrix at snapshot *t*. Without loss of generality, assume identical training symbols $s=\sqrt{P}$. By setting $∥f_{t,s}{∥}_{F}^{2}=1$, the total transmitting power is $∥f_{t,s}{s∥}_{F}^{2}=P$, and the pilot-to-noise ratio (PNR) is defined as

(20) $$\mathrm{PNR}=\frac{∥f_{t,s}{s∥}_{F}^{2}}{\sigma^{2}},$$

where the noise is assumed to be an additive white Gaussian noise (AWGN) with variance $\sigma^{2}$. In the *t*-th snapshot and after the transmitter sends out all the *S* training beams, stack the received signals as

(21) $$\begin{array}{cc} y_{t} & =\left[\begin{array}{c} f_{t,1}^{T}⊗W_{t,1}^{H}\\ f_{t,2}^{T}⊗W_{t,2}^{H}\\ \vdots \\ f_{t,S}^{T}⊗W_{t,S}^{H} \end{array}\right]\mathrm{vec}\left(H_{t}\right)+\left[\begin{array}{c} W_{t,1}^{H}n_{t,1}\\ W_{t,2}^{H}n_{t,2}\\ \vdots \\ W_{t,S}^{H}n_{t,S} \end{array}\right], \end{array}$$

(22) $$\begin{array}{c} =P_{t}\mathrm{vec}\left(H_{t}\right)+n_{t}\in C^{SK_{r}\times 1}, \end{array}$$

where

$$P_{t}=\left[\begin{array}{c} f_{t,1}^{T}⊗W_{t,1}^{H}\\ f_{t,2}^{T}⊗W_{t,2}^{H}\\ \vdots \\ f_{t,S}^{T}⊗W_{t,S}^{H} \end{array}\right],\;\mathrm{and}\;n_{t}=\left[\begin{array}{c} W_{t,1}^{H}n_{t,1}\\ W_{t,2}^{H}n_{t,2}\\ \vdots \\ W_{t,S}^{H}n_{t,S} \end{array}\right].$$

Suppose the trainings are the same for different snapshots, i.e., $f_{1,s}=f_{2,s}=\ldots =f_{T,s}=f_{s}$ and $W_{1,s}=W_{2,s}=\ldots =W_{T,s}=W_{s}$. We then have

(23) $$P=P_{1}=\ldots =P_{T}=\left[\begin{array}{c} f_{1}^{T}⊗W_{1}^{H}\\ f_{2}^{T}⊗W_{2}^{H}\\ \vdots \\ f_{S}^{T}⊗W_{S}^{H} \end{array}\right],$$

and

(24) $$\Sigma =PRP^{H}+\Sigma_{n},$$

where Σ and $\Sigma_{n}$ represent the covariance matrices of the received signal $y_{t}$ and the noise $n_{t}$, respectively.

After *T* snapshots, we can compute the dimension-reduced sample covariance matrix (SCM) of $y_{t}$ as

(25) $$S=\frac{1}{T}\sum_{t=1}^{T}y_{t}y_{t}^{H}\in C^{SK_{r}\times SK_{r}}.$$

We permute S into $S_{p}\in C^{S^{2}\times K_{r}^{2}}$ in a similar procedure as R is permuted into $R_{p}$.

### 3.4. Low-Rank Matrix Sensing Problem

We can now formulate the channel covariance estimation problem as a low-rank matrix sensing problem [25]:

(26) $$\min_{{\hat{R}}_{p}}\mathrm{rank}\left({\hat{R}}_{p}\right)\;s.t.\;{∥A\left({\hat{R}}_{p}\right)-\mathrm{vec}\left(S_{p}\right)∥}_{F}^{2}\leq \zeta^{2},$$

where ${\hat{R}}_{p}$ is the estimate of $R_{p}$, $A:C^{N_{t}^{2}\times N_{r}^{2}}\to C^{S^{2}K_{r}^{2}\times 1}$ is an appropriate linear map, and $\zeta^{2}$ is a constant to account for the fitting error. Replacing ${\hat{R}}_{p}$ with Equation (18), we can reformulate Equation (26) as

(27) $$\min_{\hat{C}}\mathrm{rank}\left(\hat{C}\right)\;s.t.\;{∥A\left(\Gamma_{u}\hat{C}\Gamma_{v}^{T}\right)-\mathrm{vec}\left(S_{p}\right)∥}_{F}^{2}\leq \zeta^{2},$$

where $\hat{C}$ is the estimate of C.

In general, problem Equation (27) is a nonconvex optimization problem and difficult to solve. In this paper, we solve the relaxed version of problem Equation (27) [26]:

(28) $$\min_{\hat{C}}\phi \left(\hat{C}\right)=f\left(\hat{C}\right)+\mu {∥\hat{C}∥}_{*}$$

where

(29) $$f\left(\hat{C}\right)=\frac{1}{2}{∥A\left(\Gamma_{u}\hat{C}\Gamma_{v}^{T}\right)-\mathrm{vec}\left(S_{p}\right)∥}_{F}^{2}$$

and μ>0 is a regularization coefficient. After some manipulations, we have $A\left(\Gamma_{u}\hat{C}\Gamma_{v}^{T}\right)=Q\mathrm{vec}\left(\hat{C}\right)$, where

(30) $$Q=\left[\begin{array}{c} \left(f_{1}^{H}⊗f_{1}^{T}\right)\Gamma_{u}⊗\left(W_{1}^{*}⊗W_{1}\right)\Gamma_{v}\\ \left(f_{1}^{H}⊗f_{2}^{T}\right)\Gamma_{u}⊗\left(W_{1}^{*}⊗W_{2}\right)\Gamma_{v}\\ \vdots \\ \left(f_{S}^{H}⊗f_{S}^{T}\right)\Gamma_{u}⊗\left(W_{S}^{*}⊗W_{S}\right)\Gamma_{v} \end{array}\right],$$

and $Q\in C^{S^{2}K_{r}^{2}\times \left(2N_{t}-1\right)\left(2N_{r}-1\right)}$. The direct evaluation of $∥\hat{C}{∥}_{*}$, which is the nuclear norm (i.e., the summation of the singular values) of $\hat{C}$, is computationally expensive. Following [21], $∥\hat{C}{∥}_{*}$ can be written as

(31) $$∥\hat{C}{∥}_{*}=\frac{1}{2}\min_{U,V}{\{∥U∥}_{F}^{2}+{∥V∥}_{F}^{2}:\hat{C}=UV^{T}\}.$$

Therefore, finding a $\hat{C}$ to minimize the objective function in Equation (28) becomes finding a pair of (U,V) to minimize

(32) $$\begin{array}{cc} \tilde{\phi }\left(U,V\right) & ≜f\left(UV^{T}\right)+\frac{1}{2}{\mu (∥U∥}_{F}^{2}+{∥V∥}_{F}^{2})\\ & =\frac{1}{2}{∥Q\mathrm{vec}\left(UV^{T}\right)-\mathrm{vec}\left(S_{p}\right)∥}_{F}^{2}\\ & +\frac{1}{2}{\mu (∥U∥}_{F}^{2}+{∥V∥}_{F}^{2}). \end{array}$$

A similar low-rank recovery problem is recently studied in [21] for instantaneous mmWave channel estimation, where a training scheme is designed such that the channel can be estimated by solving a matrix completion (MC) problem. A generalized conditional gradient and alternating minimization (GCG-Alt) algorithm is developed, which is shown to be able to provide accurate low-rank solutions at low complexity. In this work, we adapt the GCG-Alt algorithm to solve Equation (32) for our covariance matrix estimation problem. In the following, we discuss the key steps of the GCG-Alt algorithm for solving Equation (32) and refer readers to [21] for more detailed treatments.

The GCG-Alt algorithm consists of a relaxed GCG algorithm and an AltMin algorithm. Let ${\hat{C}}_{k-1}$ be the solution to C at the (k−1)-th GCG iteration. The relaxed GCG algorithm first produces an output

(33) $${\hat{C}}_{k}=\left(1-\eta_{k}\right){\hat{C}}_{k-1}+\theta_{k}Z_{k},$$

where $Z_{k}$ is the outer product of the top singular vector pair of ${\mathrm{vec}}^{-1}\left(-\nabla f\left({\hat{C}}_{k}\right)\right)$. The calculations of ${\mathrm{vec}}^{-1}\left(-\nabla f\left({\hat{C}}_{k}\right)\right)$ and the parameter $\theta_{k}$ here are different from those in [21]. For problem Equation (32), we calculate ${\mathrm{vec}}^{-1}\left(-\nabla f\left({\hat{C}}_{k}\right)\right)$ as

$$-\nabla f\left({\hat{C}}_{k}\right)=-\left(Q^{H}Q\mathrm{vec}\left({\hat{C}}_{k-1}\right)-Q\mathrm{vec}\left(S_{p}\right)\right),$$

and the parameter $\theta_{k}$ as

(34) $$\theta_{k}=\frac{R\left(q_{z_{k}}^{H}\mathrm{vec}\left(S_{p}\right)-\left(1-\eta_{k}\right)q_{z_{k}}^{H}Q\mathrm{vec}\left({\hat{C}}_{k}\right)\right)-\mu }{q_{z_{k}}^{H}q_{z_{k}}},$$

where $q_{z_{k}}=Q\mathrm{vec}\left(Z_{k}\right)$ and R(·) denotes the real part of a number. Since ${\hat{C}}_{k}=U_{k}V_{k}^{T}$, updating ${\hat{C}}_{k}$ is equivalent to updating $U_{k}=\left[\sqrt{1-\eta_{k}}U_{k-1},\sqrt{\theta_{k}}u_{k}\right]$ and $V_{k}=\left[\sqrt{1-\eta_{k}}V_{k-1},\sqrt{\theta_{k}}v_{k}\right]$. Then the obtained $U_{k}$ and $V_{k}$ are used as the initial input of the AltMin algorithm, i.e., $U_{k}^{0}\leftarrow U_{k},V_{k}^{0}\leftarrow V_{k}$. After $I_{a}$ iterations of the AltMin algorithm, update $U_{k}=U_{k}^{I_{a}}$ and $V_{k}=V_{k}^{I_{a}}$. For completeness, we summarize the GCG-Alt algorithm in Algorithm 1. After obtaining $\hat{C}$, we have ${\hat{R}}_{p}^{r}=\Gamma_{u}\hat{C}\Gamma_{u}^{T}$ and $\hat{R}=P^{-1}\left({\hat{R}}_{p}^{r}\right)$.

**Algorithm 1** The GCG-Alt Algorithm for Estimating $\hat{C}$ of Equation (28)
1:
**Input:** $\mathrm{vec}\left(S_{p}\right),Q,Q^{H}Q,\mu ,\epsilon ,\epsilon_{a}$

2:
**Initialization:** $U_{0}=⌀,V_{0}=⌀,k=0,\epsilon_{0}=\infty$

3:
**while** $\epsilon_{k}>\epsilon$ **do**
4:  $(u_{k},v_{k})\leftarrow$ singular vector pair of $Z_{k}$5:  k=k+16:  $\eta_{k}\leftarrow 2/\left(k+1\right)$ and determine $\theta_{k}$ using Equation (34);7:  $U_{k}\leftarrow \left[\sqrt{1-\eta_{k}}U_{k-1},\sqrt{\theta_{k}}u_{k}\right]$8:  $V_{k}\leftarrow \left[\sqrt{1-\eta_{k}}V_{k-1},\sqrt{\theta_{k}}v_{k}\right]$9:  **Initialization**: $i=0,\epsilon_{k}^{0}=\infty ,\left(U_{k}^{0},V_{k}^{0}\right)\leftarrow \left(U_{k},V_{k}\right)$10:  **while** $\epsilon_{k}^{i}>\epsilon_{a}$ **do**11:    i=i+112:    update $U_{k}^{i}$ and $V_{k}^{i}$ via the AltMin algorithm [21]13:    calculate $\epsilon_{k}^{i}=\frac{\tilde{\phi }\left(U_{k}^{i-1},V_{k}^{i-1}\right)-\tilde{\phi }\left(U_{k}^{i},V_{k}^{i}\right)}{\tilde{\phi }\left(U_{k}^{i-1},V_{k}^{i}\right)}$14:  **end while**15:  $\left(U_{k},V_{k}\right)\leftarrow \left(U_{k}^{i},V_{k}^{i}\right)$16:  calculate $\epsilon_{k}=\frac{\tilde{\phi }\left(U_{k-1},V_{k-1}\right)-\tilde{\phi }\left(U_{k},V_{k}\right)}{\tilde{\phi }\left(U_{k-1},V_{k-1}\right)}$17:
**end while**
18:
**Output:** $\hat{C}={\hat{C}}_{k}=U_{k}V_{k}^{T}$




### 3.5. Computational Complexity

Define a flop as an operation of real-valued numbers. Let $M=SK_{r}$ be the number of received symbols during each snapshot. Following the computational complexity analysis in [21], the computational complexity of the GCG-Alt estimator is about $8r_{\mathrm{est}}\left(I_{a}r_{\mathrm{est}}+I_{a}+1\right){\left(2N_{t}-1\right)}^{2}{\left(2N_{r}-1\right)}^{2}+8/3I_{a}r_{\mathrm{est}}\left(r_{\mathrm{est}}+1\right)\left(2r_{\mathrm{est}}+1\right)\left(2N_{t}-1\right)\left(2N_{r}-1\right)\left(N_{r}+N_{t}-1\right)+I_{a}r_{\mathrm{est}}^{2}{\left(r_{\mathrm{est}}+1\right)}^{2}\left({\left(2N_{r}-1\right)}^{3}+{\left(2N_{t}-1\right)}^{3}\right)+16r_{\mathrm{est}}\left(2N_{r}-1\right)\left(2N_{t}-1\right)M^{2}$, where $I_{a}$ is the number of iterations of the AltMin algorithm and $r_{\mathrm{est}}$ is the estimated rank of $\hat{C}$ by the GCG-Alt estimator. Later in Section 4, we show the computational complexity of the GCG-Alt estimator with specific examples.

### 3.6. Extension to the USPA System

We now follow the same process introduced in Section III. A-D to estimate the channel covariance matrix for USPA systems. To account for the different array structure of the USPA, the weight matrices of Equation (17) are redesigned. For a ${\sqrt{N}}_{a}\times {\sqrt{N}}_{a}$ USPA placed on the yz plane with distance *d* between adjacent antennas, the array response is

(35) $$a\left(\phi_{kl},\theta_{kl}\right)=a_{y}\left(\phi_{kl},\theta_{kl}\right)⊗a_{z}\left(\theta_{kl}\right),$$

where

$$a_{y}\left(\phi_{kl},\theta_{kl}\right)=\frac{1}{N_{a}^{\frac{1}{4}}}{\left[1,e^{j\frac{2\pi }{\lambda_{c}}d\sin\left(\phi_{kl}\right)\sin\left(\theta_{kl}\right)},\cdots ,e^{j\left(\sqrt{N_{a}}-1\right)\frac{2\pi }{\lambda_{c}}d\sin\left(\phi_{kl}\right)\sin\left(\theta_{kl}\right)}\right]}^{T}$$

is the array response along the *y* axis and

$$a_{z}\left(\theta_{kl}\right)=\frac{1}{N_{a}^{\frac{1}{4}}}{\left[1,e^{j\frac{2\pi }{\lambda_{c}}d\cos\left(\theta_{kl}\right)},\cdots ,e^{j\left(\sqrt{N_{a}}-1\right)\frac{2\pi }{\lambda_{c}}d\cos\left(\theta_{kl}\right)}\right]}^{T}$$

is the array response along the *z* axis. We design the weight matrices by examining the structure of ${\tilde{T}}_{kl}^{t}$ defined in Equation (3) which is written as

$$\begin{array}{cc} {\tilde{T}}_{kl}^{t} & =a_{t}^{*}\left(\phi_{kl}^{t},\theta_{kl}^{t}\right)a_{t}^{T}\left(\phi_{kl}^{t},\theta_{kl}^{t}\right)\\ & ={\left(a_{t_{y}}\left(\phi_{kl}^{t},\theta_{kl}^{t}\right)⊗a_{t_{z}}\left(\theta_{kl}^{t}\right)\right)}^{*}{\left(a_{t_{y}}\left(\phi_{kl}^{t},\theta_{kl}^{t}\right)⊗a_{t_{z}}\left(\theta_{kl}^{t}\right)\right)}^{T} \end{array}$$

where $a_{t_{y}}\left(\phi_{kl}^{t},\theta_{kl}^{t}\right)$ and $a_{t_{z}}\left(\theta_{kl}^{t}\right)$ are the transmitter array response vectors along the *y* axis and *z* axis, respectively. Note that ${\tilde{T}}_{kl}^{t}$ is block-Toeplitz–Hermitian. Let

$${\tilde{T}}_{kl}^{y}=a_{t_{y}}^{*}\left(\phi_{kl}^{t},\theta_{kl}^{t}\right)a_{t_{y}}^{T}\left(\phi_{kl}^{t},\theta_{kl}^{t}\right)\in C^{\sqrt{N_{t}}\times \sqrt{N_{t}}}$$

and

$${\tilde{T}}_{kl}^{z}=a_{t_{z}}^{*}\left(\theta_{kl}^{t}\right)a_{t_{z}}^{T}\left(\theta_{kl}^{t}\right)\in C^{\sqrt{N_{t}}\times \sqrt{N_{t}}},$$

we can verify that

(36) $${\tilde{T}}_{kl}^{t}={\tilde{T}}_{kl}^{y}⊗{\tilde{T}}_{kl}^{z},$$

and ${\tilde{T}}_{kl}^{y}$ and ${\tilde{T}}_{kl}^{z}$ are Toeplitz–Hermitian. Then for the USPA, $T_{kl}^{t}$ of Equation (8) can be written as

(37) $$T_{kl}^{t}=\frac{\gamma_{k}}{\sqrt{L}}{\tilde{T}}_{kl}^{y}⊗{\tilde{T}}_{kl}^{z}.$$

In Section III. B, we have expressed $t_{kl}^{t}={\mathrm{vec}}^{-1}\left(T_{kl}^{t}\right)$, where $T_{kl}^{t}$ of Equation (8) is Toeplitz–Hermitian matrix, in terms of a weight matrix and a vector. We have similar expressions for the vectorizations of the Toeplitz–Hermitian matrices ${\tilde{T}}_{kl}^{y}$ and ${\tilde{T}}_{kl}^{z}$. Let

(38) $$\begin{array}{cc} {\tilde{t}}_{kl}^{y} & =\mathrm{vec}({\tilde{T}}_{kl}^{y})\\ & =\Gamma_{y}a_{kl}^{y} \end{array}$$

where $\Gamma_{y}\in C^{N_{t}\times \left(2\sqrt{N_{t}}-1\right)}$ is the weight matrix and $a_{kl}^{y}\in C^{(2\sqrt{N_{t}}-1)\times 1}$, and

(39) $$\begin{array}{cc} {\tilde{t}}_{kl}^{z} & =\mathrm{vec}({\tilde{T}}_{kl}^{z})\\ & =\Gamma_{z}a_{kl}^{z} \end{array}$$

where $\Gamma_{z}\in C^{N_{t}\times \left(2\sqrt{N_{t}}-1\right)}$ is the weight matrix and $a_{kl}^{z}\in C^{(2\sqrt{N_{t}}-1)\times 1}$. Let

$$\Gamma_{y}^{\left(a\right)}={\left[\Gamma_{y}\right]}_{1+\left(a-1\right)\sqrt{N_{t}}:a\sqrt{N_{t}},:,}\;1\leq a\leq \sqrt{N_{t}},$$

and

$$\Gamma_{z}^{\left(b\right)}={\left[\Gamma_{z}\right]}_{1+\left(b-1\right)\sqrt{N_{t}}:b\sqrt{N_{t}},:},\;1\leq b\leq \sqrt{N_{t}}.$$

By exploring the matrix vectorization process, we have

(40) $$\begin{array}{cc} t_{kl}^{t} & =\mathrm{vec}(T_{kl}^{t})\\ & =\Gamma_{u}a_{kl}, \end{array}$$

where

(41) $$\Gamma_{u}=\left[\begin{array}{c} \Gamma_{y}^{\left(1\right)}⊗\Gamma_{z}^{\left(1\right)}\\ \Gamma_{y}^{\left(1\right)}⊗\Gamma_{z}^{\left(2\right)}\\ \vdots \\ \Gamma_{y}^{\left(1\right)}⊗\Gamma_{z}^{\left(\sqrt{N_{t}}\right)}\\ \Gamma_{y}^{\left(2\right)}⊗\Gamma_{z}^{\left(1\right)}\\ \Gamma_{y}^{\left(2\right)}⊗\Gamma_{z}^{\left(2\right)}\\ \vdots \\ \Gamma_{y}^{\left(\sqrt{N_{t}}\right)}⊗\Gamma_{z}^{\left(\sqrt{N_{t}}\right)} \end{array}\right]\in C^{N_{t}^{2}\times {\left(2\sqrt{N_{t}}-1\right)}^{2}}$$

is the weight matrix and

$$a_{kl}=\frac{\gamma_{k}}{\sqrt{L}}\left(a_{kl}^{y}⊗a_{kl}^{z}\right)\in C^{{\left(2\sqrt{N_{t}}-1\right)}^{2}\times 1}$$

is a vector. Then for the USPA system, $\Gamma_{u}$ of Equation (17) becomes Equation (41) and $\Gamma_{v}$ of Equation (17) is constructed similarly as Equation (41); the sizes of vectors $a_{kl}$ and $b_{kl}$ of Equation (17) have changed: $a_{kl}\in C^{{\left(2\sqrt{N_{t}}-1\right)}^{2}\times 1}$ and $b_{kl}\in C^{{\left(2\sqrt{N_{r}}-1\right)}^{2}\times 1}$, and consequently, the size for matrix C of Equation (18) has changed: $C\in C^{{\left(2\sqrt{N_{t}}-1\right)}^{2}\times {\left(2\sqrt{N_{r}}-1\right)}^{2}}$. After obtaining the weight matrices, we can follow the process in Section III. C-D to estimate C and then have the channel covariance matrix estimated as $\hat{R}=P^{-1}\left(\Gamma_{u}\hat{C}\Gamma_{v}^{T}\right)$.

## 4. Simulations

We now evaluate the performance of our proposed design for fully connected hybrid transceivers with the ULA and USPA.

### 4.1. The ULA System

We assume a carrier frequency of $f_{c}=28$ GHz. For the ULA system, $N_{t}=64,N_{r}=16,K_{t}=16$, and $K_{r}=4$. The number of clusters K∈{1,2}, and there are L=30 rays in each cluster. The horizontal AoDs and AoAs are generated as Equation (15) with $\upsilon_{h}^{t}=10.2^{\circ }$ and as Equation (16) with $\upsilon_{h}^{r}=15.5^{\circ }$, respectively. The cluster powers are generated following [22] (Table I). We compare the GCG-Alt estimator with the DCOMP estimator in [10], which has varying receiving processing matrices $W_{t,s}$ and transmitting processing vectors $f_{t,s}$ during training and has the best performance among other estimators in [10]. The DCOMP estimator needs a dictionary matrix with $G_{t}$ grid points that is associated with AoD and a dictionary matrix with $G_{r}$ grid points that is associated with AoA. Let $L_{p}$ be the number of paths in the channel, the DCOMP estimator assumes that $L_{p}$ is known. For the DCOMP estimator, we set $G_{t}=2N_{t}=128,G_{r}=2N_{r}=32$, and $L_{p}=r_{R}$. Based on Figure 2, for $p_{e}=0.99$, $r_{R}=18$ and 24 for K=1 and 2, respectively. For the GCG-Alt estimator, we set $\mu =\sigma^{2}$, ϵ=0.003, and $\epsilon_{a}=0.1$. The performance metric η [10]

$$\eta =\frac{\mathrm{tr}({\hat{M}}^{H}R\hat{M})}{\mathrm{tr}(M^{H}RM)}$$

is used to measure how close the subspace of $\hat{R}$ is to the subspace of R, where $\hat{M}\in C^{N_{t}N_{r}\times r_{R}}$ and $M\in C^{N_{t}N_{r}\times r_{R}}$ are the singular vector matrices of $\hat{R}$ and R, respectively. We also use the average of the normalized mean square error

$$\mathrm{NMSE}=\frac{∥\hat{R}{-R∥}_{F}^{2}}{{∥R∥}_{F}^{2}}$$

to measure their performance.

**Figure 3:**
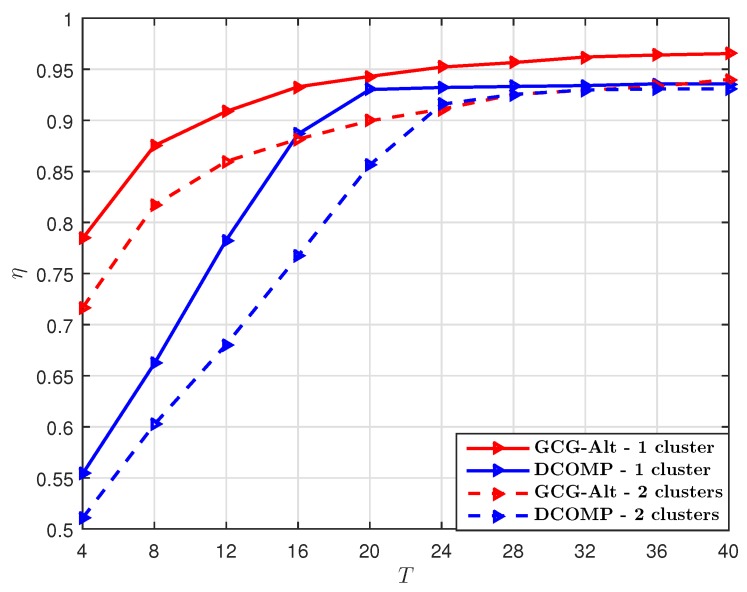
Comparison of η of the GCG-Alt estimator and the DCOMP estimator under the ULA system, where $N_{t}=64,N_{r}=16,\mathrm{PNR}=10$ dB, and S=32.

**Figure 4:**
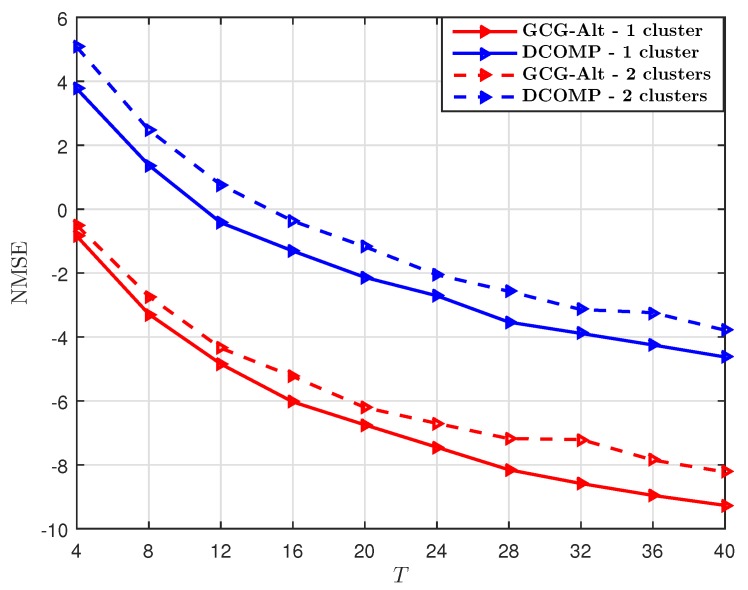
Comparison of NMSE of the GCG-Alt estimator and the DCOMP estimator under the ULA system, where $N_{t}=64,N_{r}=16,\mathrm{PNR}=10$ dB, and S=32.

We set PNR=10 dB and the number of training beams S=32, and compare the GCG-Alt estimator with the DCOMP estimator under different *T*. With S=32 per snapshot, the sampling ratio at each snapshot is $SK_{r}/N_{r}N_{t}=12.5\%$. The comparison result shown in Figure 3 suggests that when the sampling ratio per snapshot is 12.5%, our proposed estimator requires fewer snapshots to obtain an $\hat{R}$ whose subspace is close to that of R, as compared to the DCOMP estimator. The NMSE result shown in Figure 4 suggests that our proposed GCG-Alt estimator can obtain a more accurate covariance matrix estimate.

**Figure 5:**
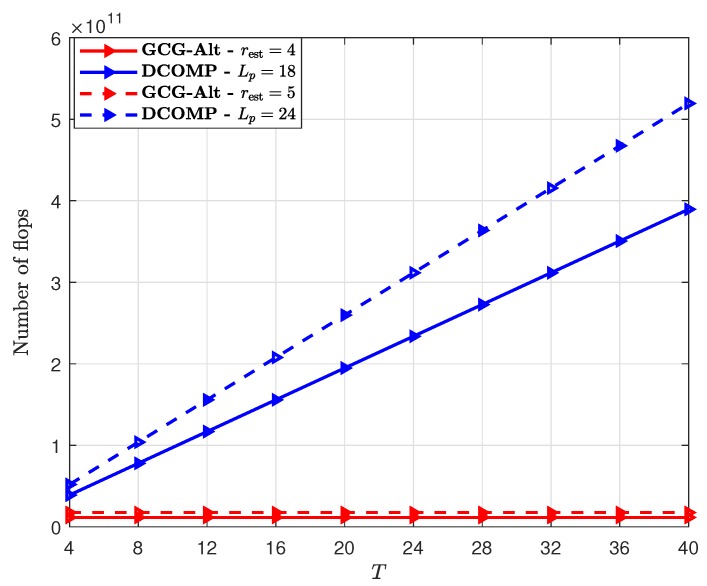
Complexity comparison of the GCG-Alt estimator and the DCOMP estimator under the ULA system, where $N_{t}=64,N_{r}=16,\mathrm{PNR}=10$ dB, and S=32.

We also compare the computational complexity of the GCG-Alt estimator and the DCOMP estimator. The computational complexity of the DCOMP estimator is about $8TL_{p}G_{t}G_{r}\left(M^{2}+M\right)$ flops, where $M=SK_{r}$. For the GCG-Alt estimator, based on our observations, the number of iterations of the AltMin algorithm $I_{a}\leq 2$, the estimated rank $r_{\mathrm{est}}\approx 4$ when K=1 and $r_{\mathrm{est}}\approx 5$ when K=2. Figure 5 shows the comparison results with different *T*. We can see that the computational complexity of the GCG-Alt estimator is lower than that of the DCOMP estimator. Also, the computational complexity of the GCG-Alt estimator does not increase as *T* increases. This is because we use $S_{p}\in C^{S^{2}\times K_{r}^{2}}$, which is the permutation of the SCM of $y_{t}$ shown in Equation (25), and its size is irrelevant to *T*.

**Figure 6:**
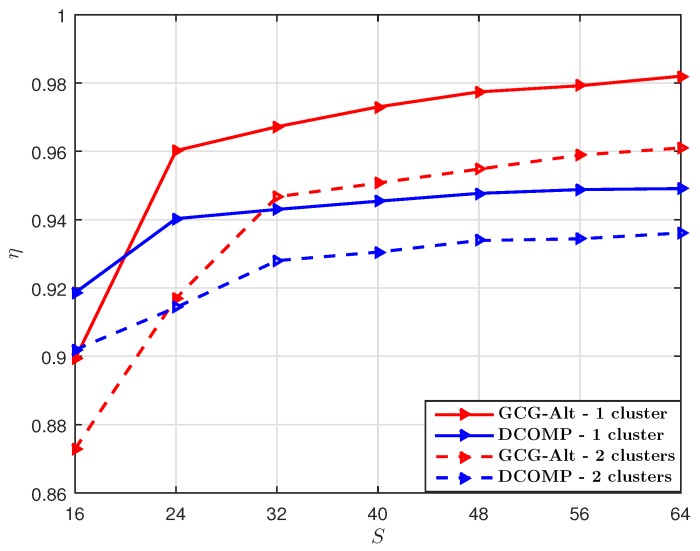
Comparison of η of the GCG-Alt estimator and the DCOMP estimator under the ULA system, where $N_{t}=64,N_{r}=16,\mathrm{PNR}=10$ dB, and T=40.

Then we set the number of snapshots T=40, and compare the GCG-Alt estimator with the DCOMP estimator under different *S*. The result shown in Figure 6 suggests that when T=40, the GCG-Alt estimator can obtain a more accurate subspace estimation than the DCOMP estimator when the number of training beams S≥24 per snapshot. Note that S=24 corresponds to a sampling ratio of 9.375% per snapshot.

The GCG-Alt estimator explores both the Kronecker structure and the block-Toeplitz–Hermitian structure of R while the DCOMP estimator only considers the Hermitian structure of R, so the GCG-Alt estimator can reach an accurate subspace estimation of R with fewer snapshots. We use the same training for different snapshots while the DCOMP estimator uses different trainings per snapshot (i.e., varying $W_{t,s}$ and $f_{t,s}$ ). When *S* is small (e.g., S≤16), the DCOMP estimator outperforms the GCG-Alt estimator. However, the GCG-Alt estimator performs better when *S* becomes larger (e.g., S≥24). Note that for the DCOMP estimator, estimating more paths (i.e., $L_{p}$ is large) yields better performance, but its computational complexity also increases.

### 4.2. The USPA System

We next consider the system with the USPA at the transmitter and receiver. The parameters $f_{c},K,L,\phi_{kl}^{t}$, and $\phi_{kl}^{r}$ are assumed the same as in the ULA system. The transmitter has an 8×8 USPA (i.e., $N_{t}=64$) and $K_{t}=16$ RF chains, and the receiver has a 4×4 USPA (i.e., $N_{r}=16$) and $K_{r}=4$ RF chains. We assume the elevation AoD angular spread $\upsilon_{v}^{t}=0^{\circ }$ and the elevation AoA angular spread $\upsilon_{v}^{r}=6^{\circ }$ based on the measurement results in [22]. The elevation AoDs and AoAs are distributed as

$$\theta_{kl}^{t}∼U\left(\theta_{k}^{t}-\upsilon_{v}^{t},\theta_{k}^{t}+\upsilon_{v}^{t}\right),$$

$$\theta_{kl}^{r}∼U\left(\theta_{k}^{r}-\upsilon_{v}^{r},\theta_{k}^{r}+\upsilon_{v}^{r}\right),$$

with the elevation center angles $\theta_{k}^{t}$ and $\theta_{k}^{r}$ being generated in the same manner as the azimuth center angles in the ULA system. For the DCOMP estimator, we set $G_{t}=2\sqrt{N_{t}}\times 2\sqrt{N_{t}}=256$ and $G_{r}=2\sqrt{N_{r}}\times 2\sqrt{N_{r}}=64$. The parameters $L_{p},\mu ,\epsilon$, and $\epsilon_{a}$ for the GCG-Alt estimator and DCOMP estimator are the same as in the ULA system.

**Figure 7:**
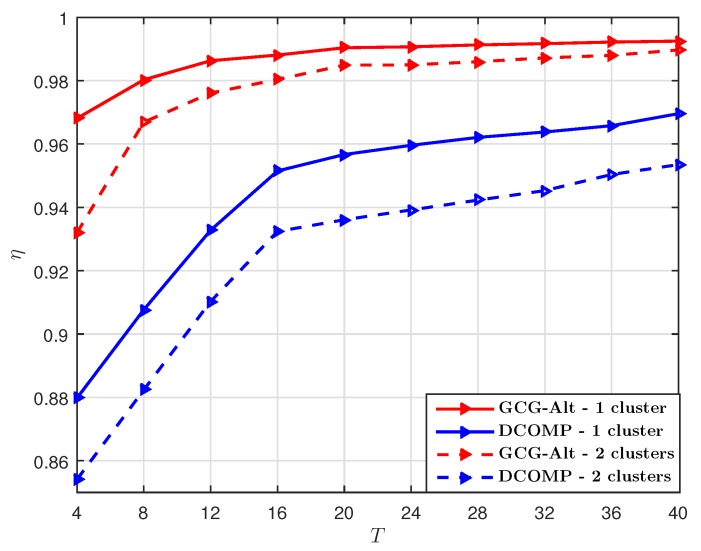
Comparison of η of the GCG-Alt estimator and the DCOMP estimator under the USPA system, where $N_{t}=64,N_{r}=16,\mathrm{PNR}=10$ dB, and S=32.

**Figure 8:**
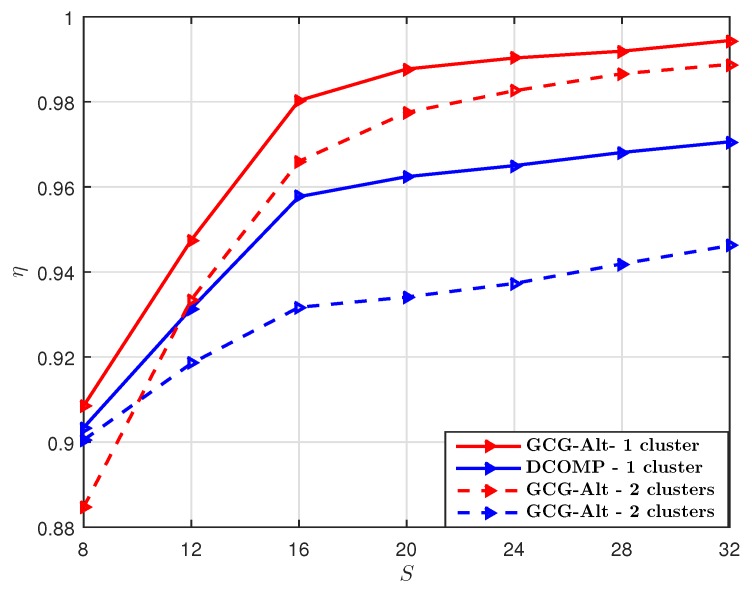
Comparison of η of the GCG-Alt estimator and the DCOMP estimator under the USPA system, where $N_{t}=64,N_{r}=16,\mathrm{PNR}=10$ dB, and T=40.

We set PNR=10 dB. The performance comparison with S=32 under different *T* is shown in Figure 7 and the performance comparison with T=40 under different *S* is shown in Figure 8. We can see that both of the GCG-Alt estimator and the DCOMP estimator achieve higher η for the USPA system. One reason for this is that the USPA system has lower resolution than the ULA system in the azimuth direction even though they have the same number of transmitter and receiver antennas. For the USPA system, the azimuth AoD is resolved by an $\sqrt{N_{t}}=8$-element antenna array and the azimuth AoA is resolved by a $\sqrt{N_{r}}=4$-element antenna array; while for the ULA system, the azimuth AoD is resolved by a $N_{t}=64$-element antenna array and the azimuth AoA is resolved by a $N_{r}=16$-element antenna array. Therefore, for the same angular spread, the USPA system resolves fewer paths than the ULA system, which results in a lower rank.

**Figure 9:**
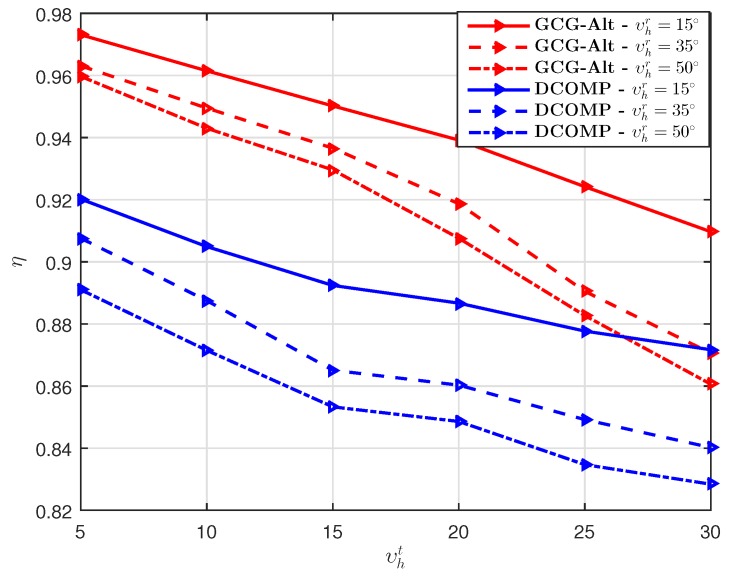
Comparison of η of the GCG-Alt estimator and the DCOMP estimator under the USPA system, where $N_{t}=64,N_{r}=16,K=1,\mathrm{PNR}=10$ dB, S=16,T=16, $\upsilon_{v}^{t}=0^{\circ }$, and $\upsilon_{v}^{r}=6^{\circ }$.

We also show the effects of angular spreads on the performance of the estimators. We set $\upsilon_{v}^{t}=0^{\circ }$, $\upsilon_{v}^{r}=6^{\circ },K=1,\mathrm{PNR}=10$ dB, S=16, and T=16. The estimators’ performance under different angular spreads for the azimuth AoD/AoA (i.e., different $\upsilon_{h}^{t}$ and $\upsilon_{h}^{r}$) shown in Figure 9 suggests that the estimators achieve lower η when $\upsilon_{h}^{t}$ and $\upsilon_{h}^{r}$ are larger.

## 5. Conclusions

We have formulated the channel covariance estimation problem for hybrid mmWave systems as a structured low-rank matrix sensing problem by exploiting Kronecker product expansion and the structures of the ULA/USPA. The formulated problem has a reduced dimensionality and is solved by using a low-complexity GCG-Alt algorithm. The computational complexity analysis and numerical results suggest that our proposed method is effective in estimating the mmWave channel covariance matrix.

## Abbreviations

The following abbreviations are used in this manuscript:

mmWave	Millimeter wave
MIMO	Multiple-input multiple-output
CSI	Channel state information
CS	Compressive sensing
AoA	Angle of arrival
AoD	Angle of departure
ULA	Uniform linear arrays
USPA	Uniform squared planar arrays
RF	Radio frequency
NMSE	Normalized mean square error
PNR	Pilot-to-noise ratio
